# Platelet transfusions in preterm infants: current concepts and controversies—a systematic review and meta-analysis

**DOI:** 10.1007/s00431-023-05031-y

**Published:** 2023-06-01

**Authors:** Helena Sofia Ribeiro, André Assunção, Rafael José Vieira, Paulo Soares, Hercília Guimarães, Filipa Flor-de-Lima

**Affiliations:** 1 Faculty of Medicine, University of Porto, Porto, Portugal; 2 Department of Pediatrics, Centro Hospitalar Universitário de São João, Porto, Portugal; 3 Department of Community Medicine, Information and Health Decision Sciences (MEDCIDS), Faculty of Medicine, University of Porto, Porto, Portugal; 4 Centre for Health Technology and Services Research, Health Research Network (CINTESIS@RISE), Faculty of Medicine , University of Porto, Porto, Portugal; 5 Department of Neonatology, Centro Hospitalar Universitário de São João, Alameda Prof Hernâni Monteiro, 4200-319 Porto, Portugal; 6 Department of Gynecology-Obstetrics and Pediatrics, Faculty of Medicine, University of Porto, Porto, Portugal

**Keywords:** Platelets, Transfusions, Preterm, Death, Sepsis, Necrotizing enterocolitis

## Abstract

Platelet transfusions (PTx) are the principal approach for treating neonatal thrombocytopenia, a common hematological abnormality affecting neonates, particularly preterm infants. However, evidence about the outcomes associated with PTx and whether they provide clinical benefit or harm is lacking. The aim of this systematic review and meta-analysis is to assess the association between PTx in preterm infants and mortality, major bleeding, sepsis, and necrotizing enterocolitis (NEC) in comparison to not transfusing or using different platelet count thresholds for transfusion. A broad electronic search in three databases was performed in December 2022. We included randomized controlled trials, and cohort and case control studies of preterm infants with thrombocytopenia that (i) compared treatment with platelet transfusion vs. no platelet transfusion, (ii) assessed the platelet count threshold for PTx, or (iii) compared single to multiple PTx. We conducted a meta-analysis to assess the association between PTx and mortality, intraventricular hemorrhage (IVH), sepsis, and NEC and, in the presence of substantial heterogeneity, leave-one-out sensitivity analysis was performed. We screened 625 abstracts and 50 full texts and identified 18 reports of 13 eligible studies. The qualitative analysis of the included studies revealed controversial results as several studies showed an association between PTx in preterm infants and a higher risk of mortality, major bleeding, sepsis, and NEC, while others did not present a significant relationship. The meta-analysis results suggest a significant association between PTx and mortality (RR 2.4, 95% CI 1.8–3.4; *p* < 0.0001), as well as sepsis (RR 4.5, 95% CI 3.7–5.6; *p* < 0.0001), after a leave-one-out sensitivity analysis. There was also found a significant correlation between PTx and NEC (RR 5.2, 95% CI 3.3–8.3; *p* < 0.0001). As we were not able to reduce heterogeneity in the assessment of the relationship between PTx and IVH, no conclusion could be taken.

*    Conclusion*: Platelet transfusions in preterm infants are associated to a higher risk of death, sepsis, and NEC and, possibly, to a higher incidence of IVH. Further studies are needed to confirm these associations, namely between PTx and IVH, and to define the threshold from which PTx should be given with less harm effect.

**Table:** 

**What is Known:** *• Platelet transfusions are given to preterm infants with thrombocytopenia either to treat bleeding or to prevent hemorrhage*. *• Lack of consensual criteria for transfusion*.	
**What is New:** *• A significant association between platelet transfusions and mortality, sepsis, and NEC*.

**Supplementary Information:**

The online version contains supplementary material available at 10.1007/s00431-023-05031-y.

## Introduction

Thrombocytopenia is a hematological abnormality commonly found in newborns, characterized by a platelet count (PC) inferior to 150 × 10^9^/L. This condition mainly affects preterm infants and critically ill neonates and it occurs due to decreased platelet production or increased consumption, or both [1]. Some common causes include fetal growth restriction (FGR), sepsis, and necrotizing enterocolitis (NEC) [1, 2]. Thrombocytopenia has been identified as a risk factor for frequent complications of prematurity, such as intraventricular hemorrhage (IVH), despite its correlation with bleeding still being uncertain [3, 4].

Platelet transfusions (PTx) remain the principal approach for treating neonatal thrombocytopenia. They are administered, generally, in two situations: to treat thrombocytopenic hemorrhage or prophylactically to prevent hemorrhage, the latter corresponding to the biggest proportion of transfusions [5, 6]. Prophylactic platelet transfusions are given to many infants even in the absence of bleeding, once their platelet counts fall below a certain threshold, aiming to prevent major hemorrhage [7, 8]. However, there is currently no consensus on the specific platelet count threshold that indicates a transfusion for a newborn [3]. These decisions are often based on physicians’ preferences, expert advice, or consensus-driven recommendations [9], and there is a lack of strong and consensual scientific evidence about whether platelet transfusions provide clinical benefit or harm to preterm infants with thrombocytopenia. Indeed, the relationship between platelet count and bleeding risk is complex in preterm infants, and the role of platelet transfusions in reducing bleeding risk is unclear [10].

This systematic review and meta-analysis aims to evaluate the risks and benefits of PTx in preterm infants compared to not transfusing or using different platelet count thresholds for transfusion. The primary outcomes in study are mortality and major bleeding (including IVH and other hemorrhage) and the secondary outcomes include sepsis and NEC.

## Methods

### Study design

We performed this systematic review and meta-analysis according to the Cochrane Handbook [11] and the Preferred Reporting Items for Systematic Reviews and Meta-Analyses (PRISMA) guidelines [12].

### Eligibility criteria

We included randomized controlled trials (RCT), cohort studies, and case control studies of preterm infants with thrombocytopenia that (i) compared treatment with platelet transfusion *versus* no platelet transfusion, (ii) assessed the platelet count threshold for PTx, or (iii) compared single to multiple PTx. We defined preterm neonates as those born before 37 weeks of gestation, and neonatal thrombocytopenia as platelet count below 150 × 10^9^/L, diagnosed during the first 28 days of life. Studies that involved both term and preterm infants were included if preterm infants represented at least 75% of the sample or if it was possible to extract separate results for preterm newborns. We considered the following outcomes: mortality of the infant or bleeding episodes post-PTx (including any grade of IVH or other hemorrhage), as primary outcomes; and morbidity, including sepsis (clinical or identified agent in hemoculture) and any stage of NEC, as secondary outcomes.

Studies including infants with congenital malformations, chromosomal anomalies, or treated with extracorporeal membrane oxygenation (ECMO) were excluded. Studies in which transfusion was not restricted to platelets were also excluded.

### Search strategy

We performed a broad electronic search in three databases, namely MedLine (via Ovid), ISI Web of Science, and Scopus, to identify relevant original articles published up to December 15, 2022, without language or date restrictions. The details of the search strategy are provided in Supplementary Information 1 (Suppl1).

### Selection process

The search results were exported to the reference management software EndNote X7, where duplicate articles were removed. The remaining articles were then uploaded to Rayyan, a web tool used for systematic reviews, and any lasting duplicates were excluded.

Two reviewers independently screened the articles initially by titles and abstracts and disagreements were resolved by consensus. The full-text versions of the articles that passed the initial screening were retrieved and assessed for eligibility by the same two reviewers, independently. Any disagreements were resolved by consensus and, if necessary, a third reviewer was consulted for discussion.

### Data extraction and critical appraisal of individual studies

Data extraction was accomplished by two researchers using a purposely built Excel form. The following information was extracted from each study: author, year of publication, study design; number of preterm newborns, gender, gestational age, and birth weight of the total sample and in each group; available data about the intervention (total and mean number of transfusions, threshold, and PTx criteria per group); and the incidence of the outcomes: mortality, IVH, other bleeding events, sepsis, and NEC. Two tables were created based on the study objectives: one for studies that compared receiving vs. not receiving PTx and another for studies comparing different thresholds of transfusion or single with multiple PTx.

To assess the quality of the studies and the potential sources of bias, we used the Cochrane risk-of-bias tool, version 2 [11], for randomized controlled trials and the Newcastle–Ottawa Scale (NOS) for observational studies. The 9-point NOS is divided into three main categories: selection, comparability, and exposure [13]. Studies that scored a total of 7–9 stars were considered of “good quality,” 4–6 of “fair quality,” and 0–3 of “poor quality.”

### Data synthesis

We conducted meta-analysis assessing the association between PTx and mortality, IVH, sepsis, and NEC based on relative risks (RR) retrieved or calculated from included primary studies. We used a random-effects model weighting for the restricted maximum likelihood approach to estimate the pooled RRs. In each meta-analysis, only primary studies with the same design were included (i.e., we did not include observational and experimental studies in the same analysis). Inter-study heterogeneity was assessed using the *I*^2^ statistic and the Cochran *Q* statistic. An *I*^2^ > 50% and a Cochran *Q* statistic* p* value less than 0.1 were considered to represent substantial heterogeneity and, in the presence of substantial heterogeneity, leave-one-out sensitivity analysis was conducted. Data analysis was performed using software R (version 4.0), with use of meta package.

## Results

### Study selection

Our search returned a total of 1100 articles. After removing duplicates, 625 reports were screened by title and abstract, leading to the exclusion of 573 articles. The remaining 50 full-text articles retrieved were thoroughly analyzed for eligibility, and 18 reports from 13 studies met the inclusion criteria and were ultimately included in this review. Suppl2 illustrates the screening and selection process.

### Overview of the included studies

Our review includes 10 reports [14–23] from 8 studies that assessed the effects of PTx in preterm infants, comparing transfused infants vs. non-transfused infants. The characteristics of each study (population and interventions) are presented in Suppl3, and the corresponding outcomes are listed in Table 1. We included 5 additional reports [24–28] from 3 studies that compared different transfusion thresholds and 3 reports [29–31] from 2 studies comparing single vs. multiple transfusions. Table 2 provides demographic and intervention information for each study, while Table 3 outlines the assessed outcomes. A total of 7 reports from the 3 RCT conducted on this topic were included [14–16, 25–28].

**Table 1:** Outcomes studied and comparison between transfused and non-transfused groups

**Author, year**	***N***	***n***	**Mortality** ***n* (%)**			**IVH** ***n* (%)**		**Other hemorrhage** ***n* (%)**		**Sepsis** ***n* (%)**		**NEC** ***n* (%)**		**Findings**
		**G1**	**G2**	**G1**	**G2**	**G1**	**G2**	**G1**	**G2**	**G1**	**G2**	**G1**	**G2**	
**Andrew** **1993 **[14–16]	152	78	74	16(20.5)	11(14.9)	-	-	22(28.2) ^a^	19(25.7) ^a^	-	-	3 (3.8)	1 (1.4)	No association between PTx and intracranial hemorrhage (*p* = 0.73)
**Christensen 2006 **[17]	283	129	154	29(22.5)	18(11.7)	-	-	-	-	-	-	-	-	Significant association between PTx and mortality rate (*p* < 0.01)
**Baer 2007 **[18]	1600	494	1106	81(16.4)	22(2.0)	99(20.0)^b^	44(4.0^b^	-	-	143(28.9)	77(7.0)	54(10.9)	22(2.0)	Significant association between PTx and mortality and greater increase with a higher number of transfusions (*p* < 0.001) Significant association between PTx and IVH (*p* < 0.001)
**Bonifacio 2007 **[19]	82	60	22	29(48.3)	4(18.2)	37(61.7)	7(31.8)	-	-	34(56.7)	10(45.5)	-	-	No association between PTx and mortality, IVH, or sepsis
**Sparger** **2016 **[20]	972	212^c^	190^c^	-	-	97(45.8)^c^	39 (20.5)^c^	42 (19.8)^c,d^	6 (3.2)^c,d^	-	-	-	-	Significant association between PTx and IVH (*p* = 0.005) and other bleeding (*p* < 0.001) After adjusting for relevant variables, no significant association between PTx and IVH
**Alhamad 2022 **[21]	154	77	77	30(39.0)	15(19.5)	59(76.6)	71(92.2)	-	-	-	-	1 (1.3)	0 (0.0)	No association between PTx frequency and threshold and mortality rate
**Chen 2002 **[22]	1221	94	1127	18(19.1)	62 (5.5)	50(53.2)	213(18.9)	-	-	16(17.0)	35 (3.1)	1 (1.1)	5 (0.4)	Significant association between PTx and mortality (HR 1.48; 95%CI 1.13–1.93; *p* = .004) No association between PT and IVH
**Raja 2022 **[23]	540	105	435	-	-	14(13.3)^b^	7 (1.6) ^b^	-	-	-	-	-	-	Significant association between PTx and all grades of IVH (OR: 4.3, 95% CI: 2.3–8.2) and severe IVH (OR: 41, 95% CI: 14.0–146.6)

*n* number of infants, *IVH* Intraventricular Hemorrhage, *NEC *Necrotizing Enterocolitis, *G1* Group 1—received platelet transfusion, *G2* Group 2—did not receive platelet transfusion *PTx* Platelet Transfusion, *HR* Hazard Ratio, *CI* Confidence Interval, *OR* Odds Ratio -, outcomes not studied

^a^Intracranial hemorrhage

^b^Only grade 3 or 4 IVH

^c^Number in patient-days, during the first 7 days of life with a platelet count < 100 × 109/L, in whom the outcomes were studied. Absolute numbers of patients in each group: Group 1, 231; Group 2, 741

^d^Pulmonary, gastrointestinal, genitourinary, wound, or other hemorrhage (not including IVH)

**Table 2:** Demographic and intervention data in studies comparing preterm infants who received platelet transfusions from different platelet count thresholds or different numbers of platelet transfusions

**Author, year**	**Study design**	***N***	**Group description**	***n***	**Male** ***n* (%)**	**Gestational age, weeks** **(mean ± SD)**	**Birth weight, grams** **(mean ± SD)**	**Intervention details**
Garcia 2001 [29, 30]	Retrospective cohort	61	**G1:** Received more than 1 PTx	54	37 (68.5)	32.6 ± 3.7	1720 ± 752	Criteria for PTx: 1. PC < 20 × 10^9^/L 2. PC < 50 × 10^9^/L with active bleeding
			**G2:** Received only 1 PTx	7	6 (85.7)	33.6 ± 6.2	1675 ± 1052	
**Del Vecchio 2001 **[31]	Retrospective cohort	114	**G1:** Received more than 1 PTx	59	29 (49.2)	30.1 ± 5.8	1408 ± 990	Total number of transfusions: 371
			**G2:** Received only 1 PTx	55	21 (38.2)	30.8 ± 5.2	1568 ± 943	
**von Lindern 2012 **[24]	Retrospective cohort	679	**G1:** Liberal transfusion group	326	172 (52.8)	28.9 ± 1.9	1259 ± 326	Criteria for PTx: 1. PC < 30 × 10^9^/L in clinically stable infants 2. PC < 50 × 10^9^/L in unstable infants, before planned surgery, or both 3. < 100 × 10^9^/L in cases of active bleeding, at the start of an exchange transfusion or both Mean number of transfusions ± SD: 1.1 ± 3.0
			**G2:** Restrictive transfusion group	353	195 (55.2)	28.9 ± 1.9	1270 ± 353	Criteria for PTx: PC < 50 × 10^9^/L in the presence of: - Major bleeding, massive petechiae, and bruising - Need for surgery, invasive procedures, or indomethacin treatment for PDA Prophylactic PTx not given if infants had severe thrombocytopenia but no clinical signs of bleeding Mean number of transfusions ± SD: 0.2 ± 0.7
**Curley** **2019 **[25–27]	RCT	660	**G1:** Liberal transfusion group	329	205 (62.3)	26.6 (24.9–28.9) ^a^	728 (600–940) ^a^	PTx given at PC thresholds of 50 × 10^9^/L 90% of the infants (296) received at least 1 PTx
			**G2:** Restrictive transfusion group	331	191 (57.7)	26.7 (24.9–28.7) ^a^	743 (605–990) ^a^	PTx given at PC thresholds of 25 × 10^9^/L 53% of the infants (177) received at least 1 PTx
**Kumar** **2019 **[28]	RCT	44	**G1:** Liberal transfusion group	22	11 (50.0)	29.3 ± 2.4	1075 ± 308	Infants received PTx to maintain PC > 100 × 10^9^/L until one of the following: 1. PDA closed 2. 120 h after randomization
			**G2:** Restrictive transfusion group	22	13 (59.1)	30.0 ± 2.0	1149 ± 303	Standard criteria for transfusion: 1. PC < 20 × 10^9^/L 2. Clinical bleed 3. PC < 50 × 10^9^/L + requiring a major non-neurosurgical procedure 4. PC < 100 × 10^9^/L + requiring a neurosurgical procedure

*N* Total number of infants in the study, *n* number of infants per group, *SD* Standard Deviation, *G1* Group 1, *G2* Group 2, *RCT* Randomized Controlled Trial, *PTx* Platelet Transfusion, *PC* Platelet Count, *PDA* Patent Ductus Arteriosus

^a^Median (interquartile range)

**Table 3:** Outcomes studied and comparison between groups who received platelet transfusions from different platelet count thresholds or different numbers of platelet transfusions

**Author, year**	***N***	***n***		**Mortality** ***n* (%)**		**IVH** ***n* (%)**		**Other hemorrhage** ***n* (%)**		**Sepsis** ***n* (%)**		**NEC** ***n* (%)**		**Findings**
		**G1**	**G2**	**G1**	**G2**	**G1**	**G2**	**G1**	**G2**	**G1**	**G2**	**G1**	**G2**	
Garcia 2001 [29, 30]	61	54	7	12 (22.2)	3 (42.9)	21 (38.9)	4 (57.1)	-	-	24 (44.4)	4 (57.1)	10 (18.5)	0 (0.0)	No association between the increasing number of PTx given and mortality
**Del Vecchio 2001 **[31]	114	59	55	†	†	-	-	-	-	-	-	-	-	Significant increase in mortality rate in infants who received > 4 PTx vs. those who received 1 PTx (RR 2.9; *p* < 0.0001). Significant increase in infants who received 1 PTx vs. all the other infants in the NICU who did not receive any (RR 10.4; *p* < 0.0001)
**von Lindern 2012 **[24]	679	326	353	22 (6.7)	25 (7.1)	63 (19.5) ^a^	75 (22.7) ^a^	-	-	98 (30.1)	89(25.2)	9(2.8)	14(4.0)	No significant difference in the incidence of IVH between liberal and restrictive transfusion groups (*p* = 0.31)
**Curley 2019** [25–27]	660	329^b^	331^b^	**G1:** 85(26.2)^*c*^ **G2:** 61 (18.5)^*c*^						181(55.9)	175(53.7)^d^	42(13.0)	54 (16.6)^d^	Significant increase in mortality and major bleeding in preterm infants in the liberal transfusion group vs. restrictive group (OR 1.57; 95%CI 1.06 -2.32; *p* = 0.02) No significant differences in sepsis and NEC between groups
**Kumar 2019 **[28]	44	22	22	8 (36.4)	9 (40.9)	9 (40.9)	2 (9.1)	-	-	8 (36.3)	2 (9.1)	-	-	Significant association between liberal transfusion and any grade of IVH vs. restrictive transfusion (*p* = 0.034) No significant differences between liberal and restrictive transfusion groups in grade III or IV IVH or mortality

*n *number of infants, *IVH* Intraventricular Hemorrhage, *NEC* Necrotizing Enterocolitis, *G1* Group 1, *G2* Group 2, *PTx* Platelet Transfusion, *RR* relative Risk, *NICU* Neonatal Intensive Care Unit, *OR*, Odds Ratio, *CI* Confidence Interval, -, outcomes not studied, † outcome studied but unavailable number and percentage of infants who developed the outcome

^a^Percentage calculated for a total number of infants who had a cranial ultrasound scan: 323 infants in group 1 and 330 in group 2

^b^5 infants in group 1 and 2 infants in group 2 did not have primary outcomes reported and were excluded in the phase of outcomes analysis due to palliative care (3); randomization error (2); and issues with consenting process (2): Group 1, 324; Group 2, 329

^c^Death and major bleeding were accessed together, as the primary outcome of the study

^d^Percentage calculated for a total number of 326 infants in group 2

To perform the meta-analyses, only studies comparing PTx vs. non-transfusion were included [17–19, 21, 22]. Some studies comparing transfused vs. non-transfused infants were not included in the meta-analyses: Andrew et al. [14–16] for being the sole RCT; Sparger et al. [20] because outcome values were presented in patient-days; Raja et al. [23] and Del Vecchio et al. [31] due to the unavailability of number and percentage of infants with outcome events. In addition, studies assessing liberal vs. restrictive transfusion or different number of PTx were excluded due to methodological heterogeneity.

No studies assessing the association between PTx and retinopathy of prematurity (ROP), patent ductus arteriosus (PDA), or bronchopulmonary dysplasia (BPD) met the inclusion criteria or had extractable data.

### Risk of bias assessment

The three RCT included in the analysis were evaluated for bias risk and the two most recent trials [25–28] showed a low risk, while that of Andrews et al. [14–16] was found to have some potential risk of bias, due to deviations from the intervention in the control group, in case of major bleeding or platelet count below 50 × 10^9^/L.

Concerning the ten observational studies included, seven [18–22, 24, 31] scored 7 stars or higher, being classified as “good quality,” while three [17, 23, 29, 30] scored 4 to 6 stars and were categorized as “fair quality.”

Detailed assessments of the risk of bias within each study are presented in Supplementary Information: Suppl4 for RCT and Suppl5 for observational studies.

### Mortality

**Fig. 1:**
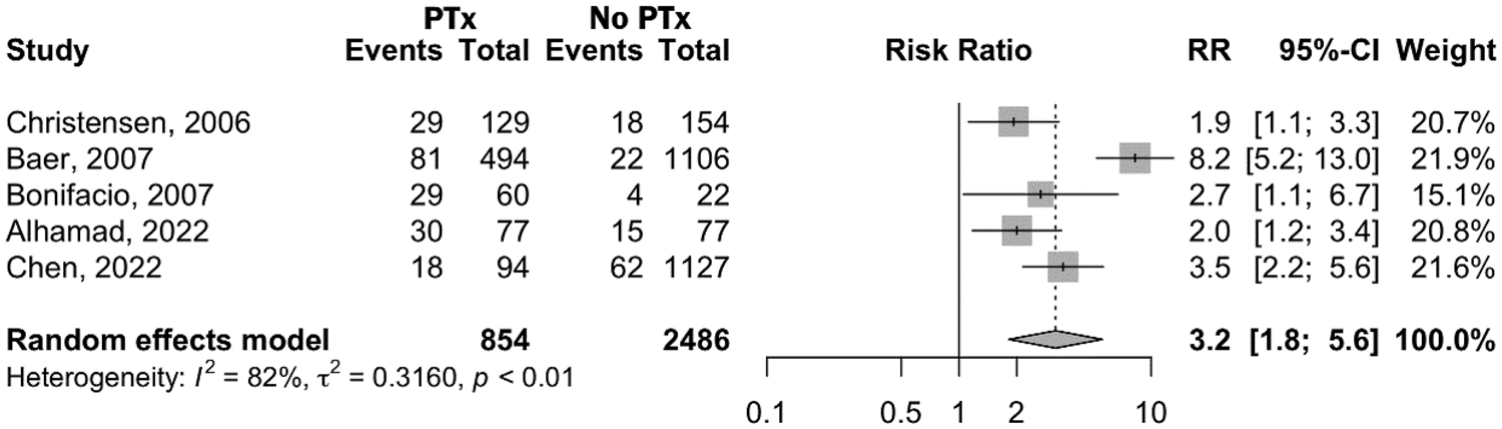
Forest plot comparing mortality in preterm infants who received platelet transfusions and those who did not receive platelet transfusions

Among the studies that compared preterm infants who received PTx with those who did not, five [17–19, 21, 22] assessed the relationship between receiving PTx and mortality. Three studies [17, 18, 22] found a significant increase in mortality rate in the transfused group in comparison to the non-transfused one. In contrast, two studies [19, 21] did not find a significant association between PTx and mortality, even though the mortality rate was higher among patients who received PTx. The forest plot in Fig. 1 illustrates the comparison between transfused and non-transfused groups. The results show a significantly higher mortality in the PTx group [relative risk (RR) 3.2, 95% confidence interval (CI) 1.8–5.6; *p* < 0.0001] than in the non-transfused group. Significant heterogeneity was present across the studies (*I*^2^ = 82%, *p* < 0.01), so a leave-one-out sensitivity analysis was conducted to identify potential sources of heterogeneity in the quantitative analysis. Regardless of which study is removed, there is always a significantly higher risk of death in the transfused group (Suppl6). Heterogeneity decreases substantially to 12% when Baer et al. [18] is removed, indicating that this study is the primary source of heterogeneity. Upon omitting this study, the risk of death is 2.4 times higher in the transfused group vs. the non-transfused one (95% CI 1.8–3.4; *p* < 0.0001).

Regarding the evaluation of different transfusion thresholds, PlaNeT-2 Trial [25–27] found a significant increase in mortality and major bleeding in the liberal transfusion group when compared to the restrictive approach. Likewise, Del Vecchio et al. [31] reported that the risk of death was significantly higher in patients who receive more than four transfusions vs. those who receive solely one or no PTx. Kumar et al. [28], however, did not observe a higher mortality rate in the liberal PTx group vs. the restrictive one. Garcia et al. [29; 30] also did not identify an association between giving multiple PTx to preterm infants and a higher mortality rate in comparison to solely one platelet transfusion.

### Intraventricular hemorrhage and other bleeding events

**Fig. 2:**
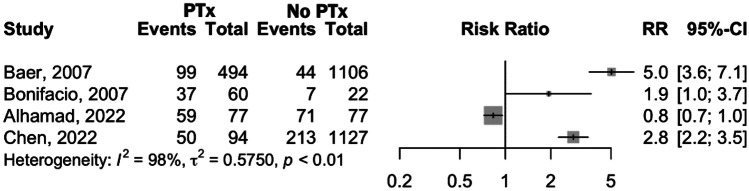
Forest plot comparing IVH incidence in preterm infants who received platelet transfusions and those who did not receive platelet transfusions

The influence of PTx in the occurrence of new-onset IVH was studied in five articles [18–20, 22, 23] that compared the outcome in transfused and non-transfused infants. While two studies [19, 22] did not find a significant correlation between PTx and IVH, Baer et al. [18] observed a significant increase of IVH events in transfused newborns. Similarly, Sparger et al. [20] found a significant association, not only for IVH but also for other bleeding events; however, after two multivariable regression models, the relationship weakened and was no longer significant. In contrast, even after adjusting for possible confounders, in Raja et al. [23], PTx were found to be a significant predictor of severe IVH (*p* < 0.001), with odds ratio (OR) of 4.3 for developing any grade of IVH (95% CI: 2.3–8.2) and 41 for grade III or IV (95% CI 14.0–146.6). Andrew et al. [14–16] evaluated the incidence of intracranial hemorrhage (ICH) and did not find a correlation between PTx and ICH. Quantitative assessment for IVH is shown in Fig. 2 and because of the high degree of heterogeneity observed (*I*^2^ = 98%, *p* < 0.01), which was not resolved by leave-one-out sensitivity analysis, the meta-analytical effect measure is not presented.

In relation to liberal vs. restrictive transfusion, the two RCT [25–28] reported a significantly higher incidence of IVH in the liberal transfusion group but von Linder et al. [24] did not observe differences between groups.

### Morbidity: sepsis and necrotizing enterocolitis

None of the studies included in this systematic review had the primary objective of assessing the effects of PTx on sepsis, NEC, or other newborn diseases besides bleeding. Although several studies provide data on the association between PTx and sepsis, only Bonifacio et al. [19] and Curley et al. [25–27] performed statistical analyses, reporting a non-significant correlation between PTx and sepsis, and liberal transfusion and sepsis, respectively. With regard to NEC, Curley et al. [25–27] also did not observe a significant relationship.

**Fig. 3:**
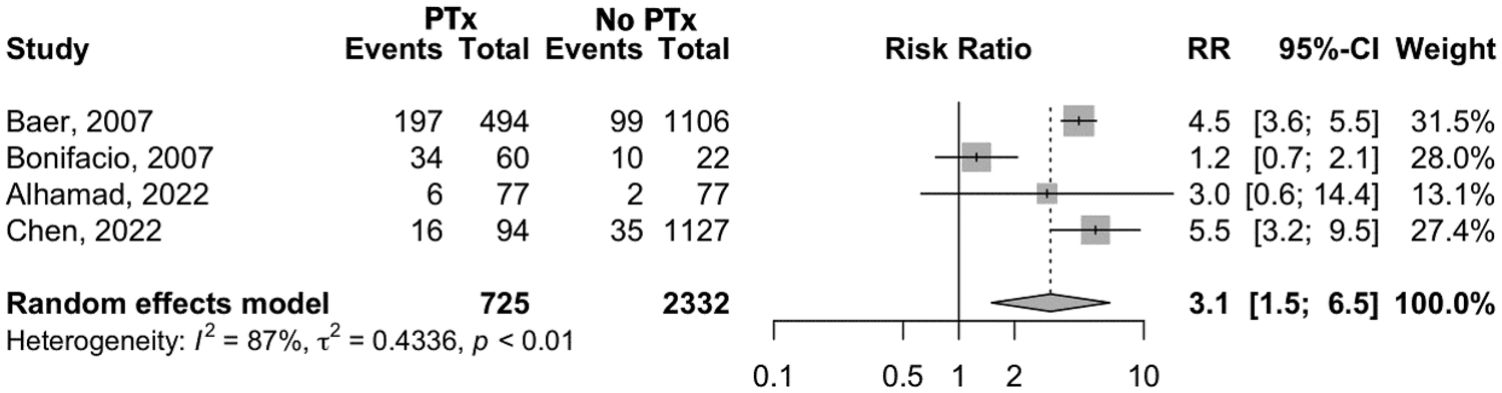
Forest plot comparing sepsis incidence in preterm infants who received platelet transfusions and those who did not receive platelet transfusions

Despite that, we conducted a meta-analysis for both outcomes using data present in several studies [18, 19, 21, 22]. The forest plot for sepsis in presented in Fig. 3 that shows a significantly higher incidence in PTx-receiving group than in the non-transfused group [RR 3.1, 95% CI 1.5–6.5; *p* < 0.0001]. Due to significant heterogeneity across the studies (*I*^2^ = 87%, *p* < 0.01), a leave-one-out sensitivity analysis was conducted (Suppl7) and, when omitting Bonifacio et al. [19], heterogeneity was no longer present and a significant association between PTx and sepsis remained (RR 4.5, 95% CI 3.7–5.6; *p* < 0.0001).

**Fig. 4:**
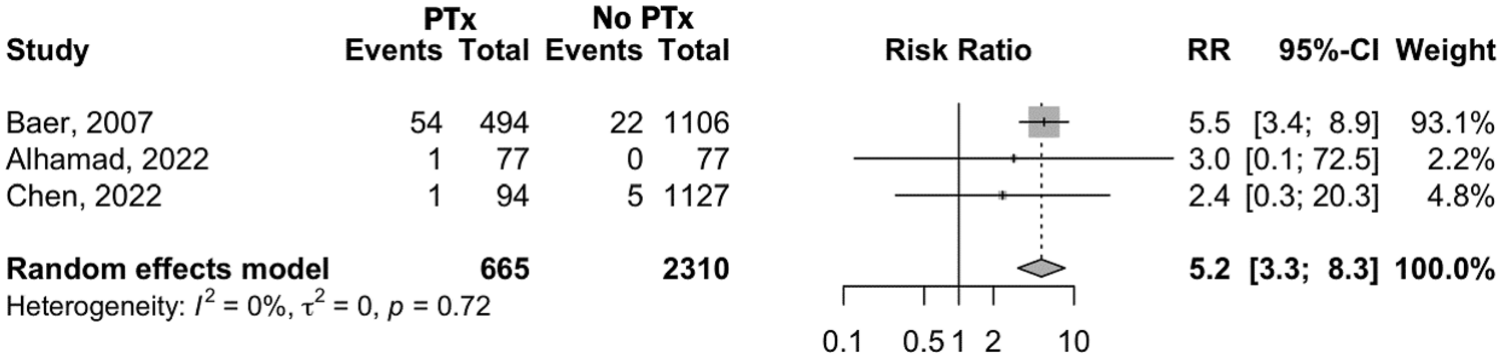
Forest plot comparing NEC incidence in preterm infants who received platelet transfusions and those who did not receive platelet transfusions

The forest plot for NEC, illustrated in Fig. 4, shows that the risk of developing NEC in the PTx group was significantly higher than in the non-transfused group (RR 5.2, 95% CI 3.3–8.3; *p* < 0.0001), with no heterogeneity detected (*I*^2^ = 0%; *p* = 0.72).

## Discussion

The qualitative analysis of the studies included in this systematic review has revealed the controversial nature of the topic and the lack of consistent evidence about the effects of PTx in preterm infants. In fact, several studies have shown an association between PTx in thrombocytopenic preterm infants and a higher risk of mortality, major bleeding, sepsis, and NEC, while others did not show a significant relationship. The same happens with studies assessing different thresholds (i.e., the deleterious effect of transfusion at higher thresholds). These discrepancies may be partly attributed to methodological differences across the studies (e.g., demographic characteristics, interventions applied, and measured outcomes). These methodological disparities pose challenges when comparing the studies and drawing definitive conclusions.

Our meta-analysis aimed to assess the potential relationships between PTx and the outcomes in study. Despite the presence of high heterogeneity, significant risk-associations were found between PTx and adverse outcomes. A leave-one-out sensitivity analyses for mortality and sepsis outcomes allowed for decreased heterogeneity. Motives of such heterogeneity are already mentioned above. Based on these results, PTx in preterm infants are associated to increased risk of mortality, sepsis, and NEC. Evidence regarding the association between PTx and IVH is too heterogeneous and a meta-analysis could not be conducted.

### Mortality and intraventricular hemorrhage and other bleeding events

The results related to mortality are in agreement with those of the largest RCT conducted to date on the effects of different transfusion thresholds in mortality and the development of major bleeding. The PlaNeT-2 trial [25–27] demonstrated that administering prophylactic PTx at a lower threshold (PC below 25 × 10^9^/L) resulted in lower mortality rates and hemorrhage episodes in infants than transfusions at a higher threshold (PC below 50 × 10^9^/L). The liberal transfusion group received more PTx and had more adverse events than the restrictive one. Another RCT was conducted by Kumar et al. [28], which not only aimed principally to assess the relation between PTx and patent ductus arteriosus (PDA), but also found that IVH events were significantly higher in the liberal transfusion group.

It is well-known that platelets have an imperative role in primary hemostasis. However, in newborn infants, particularly those born prematurely, platelets tend to be less active, which can result in longer bleeding and clotting times compared to healthy neonates born at full-term [5]. As preterm infants have a greater likelihood of bleeding, especially IVH, neonatologists frequently administer platelet transfusions in thrombocytopenic newborns without bleeding signs in an attempt to prevent bleeding and reduce the risk of further complications [32]. Unfortunately, and despite the theoretical benefit that PTx should have, no trials have yet conclusively demonstrated a protective effect of PTx on bleeding risk in preterm neonates with severe thrombocytopenia [2]. In fact, the studies reviewed in this analysis showed either a higher or similar risk of adverse effects between the transfused vs. non-transfused group but never an improvement in the health outcomes of the transfused group because of the intervention. The mechanisms underlying the deleterious effects of PTx are still unknown but some possible explanations are the use of transfusion products from adult donors, whose platelets are hyperreactive and pro-inflammatory in comparison to newborns’ ones and also the rapid expansion of blood volume due to the PTx, which can be the cause of bleeding [33].

As PTx can result in unfavorable consequences, it is still unclear what specific threshold provides a greater benefit over risk. Curley et al. [25–27] and Kumar et al. [28] suggest that a lower threshold should be used. However, further randomized controlled trials are necessary to determine the exact PC threshold at which transfusion is beneficial and should be performed. This will help prevent harm to newborns, avoid unnecessary costs, and prevent overuse of blood bank resources [5].

### Morbidity

Sepsis and NEC were the two outcomes in study concerning morbidity of the newborn because sufficient data could be extracted from the included articles for comparison. However, there was limited data available on the outcomes of ROP, PDA, and BPD, so these outcomes were not included in the analysis. Further research to assess the consequences of PTx in the occurrence of these outcomes would be useful.

### Strengths and limitations

Our review has several strengths, including a broad search strategy, as well as the inclusion of both RCTs and observational studies, which have an adequate risk of bias assessment, strengthening the results obtained. We also present and compare the results of studies with different objectives: transfusion vs. no transfusion; different transfusion thresholds and single vs. multiple transfusions. To our knowledge, it is the first meta-analysis conducted on this specific topic.

One limitation of this review is the retrospective nature of the majority of non-randomized studies, which are generally considered to be of lower quality compared to RCT. However, we still included these studies as they can provide valuable insights. To address this potential limitation, we conducted a risk of bias analysis as part of our study methodology. Another limitation is that temporal ambiguity presents a complex challenge, making it difficult to establish causality between outcomes and PTx, as opposed to the underlying thrombocytopenia. In addition, the fragility of the preterm population and the presence of confounding factors, such as comorbidities associated with newborns, make it difficult to draw definitive conclusions because there are events that cannot be fully eliminated when studying humans and, particularly, neonates. For example, common causes of thrombocytopenia, like sepsis and NEC [1; 2], may be a consequence of PTx rather than the primary cause. Likewise, it is possible that a higher number of PTx are administered due to the presence of IVH or other hemorrhage, rather than bleeding being caused by the transfusions. To minimize these confounding effects, in every included study, we ensured that the outcomes were not present before the intervention. However, the non-randomized design of some studies introduces the potential for selection bias, whereby infants who received PTx may have had higher levels of morbidity, which could have influenced the occurrence of adverse outcomes. Additionally, there is missing data from the original studies regarding the index of severity of illness and causes of death. Furthermore, the meta-analyses’ results may be limited by the relatively small number of studies that met the inclusion criteria, as well as their heterogeneity. A Leave-one-out sensitivity analyses for mortality and sepsis showed a decrease in heterogeneity without affecting our meta-analytical results, which supports the robustness and reliability of our findings. A meta-regression could not be done due to the small number of studies.

## Conclusion

Platelet transfusions in preterm infants are associated to a higher risk of death, sepsis, and NEC and, possibly, to a higher incidence of IVH. Further studies are needed to confirm these associations, namely between PTx and IVH, and to define the threshold from which PTx should be given with less harmful effect. Preferably, RCTs should be conducted in preterm infants, comparing the outcomes of transfused and non-transfused patients at defined and comparable thresholds. Furthermore, it would be valuable to assess outcomes based on the number of transfusions received.

## Supplementary Information

Below is the link to the electronic supplementary material.

Supplementary file1 (DOCX 401 KB)

## Abbreviations

BPD: Bronchopulmonary dysplasia
CI: Confidence interval
ECMO: Extracorporeal membrane oxygenation
FGR: Fetal growth restriction
*I*^2^: I-square
ICH: Intracranial hemorrhage
ISI: Institute of Scientific Information
IVH: Intraventricular hemorrhage
NEC: Necrotizing enterocolitis
NOS: Newcastle-Ottawa Scale
OR: Odds ratio
PC: Platelet count
PDA: Patent ductus arteriosus
PRISMA: Preferred Reporting Items for Systematic Reviews and Meta-Analyses
PTx: Platelet transfusions
RCT: Randomized controlled trial
ROP: Retinopathy of prematurity
RR: Relative risk
