# Supplementary material for: Platelet transfusions in preterm infants: current concepts and controversies—a systematic review and meta-analysis
Source: Eur J Pediatr. 2023 Jun 1;182(8):3433–43. doi: 10.1007/s00431-023-05031-y (PMC10460362; doi:10.1007/s00431-023-05031-y)
Supplement: Supplementary file 1 — Supplementary file1 (DOCX 401 KB) [file 431_2023_5031_MOESM1_ESM.docx]

**Supplementary Information**

**Suppl1: Search strategy details**

| **Medline via OVID** | |
| --- | --- |
| **Number** | **Query** |
| #1 | BLOOD PLATELETS/ |
| #2 | (platelet* or thrombocyte*).ti. |
| #3 | #1 OR #2 |
| #4 | exp BLOOD TRANSFUSION/ |
| #5 | ransfuse*.ti. |
| #6 | #4 OR #5 |
| #7 | #3 AND #6 |
| #8 | PLATELET TRANSFUSION/ |
| #9 | PLATELETPHERESIS/ |
| #10 | ((platelet* or thrombocyte*) adj5 (prophyla* or transfus* or infus* or administ* or requir* or need* or product* or component* or concentrate* or apheres* or pooled or single donor or random donor)).tw. |
| #11 | (thrombocytopheres* or plateletpheres*).tw. |
| #12 | ((platelet* or thrombocyte*) adj5 (protocol* or trigger* or threshold* or schedul* or dose* or dosing or usage or utili?ation)).tw. |
| #13 | #7 OR #8 OR #9 OR #10 OR #11 OR #12 |
| #14 | Infant, Premature/ or Infants, Extremely premature/ or Infant, Low birth weight/ or Infant, Very Low Birth Weight/ or Intensive care units, Neonatal/ or Intensive care, Neonatal/ or Premature birth/ |
| #15 | (((low birthweight or low birth weight or VLBW or ELBW or Prematur* or Preterm or Pre-term) adj2 (Infant* or Neonat* or newborn* or new-born* or baby* or babies)) or neonatal intensive care or NICU).tw,kw. |
| #16 | #14 OR #15 |
| #17 | #13 AND #16 |
| **ISI Web of Science** | |
| **Number** | **Query** |
| #1 | TI=((platelet* OR thrombocyte*) AND transfus*) |
| #2 | TS=((platelet* OR thrombocyte*) NEAR/5 (prophyla* OR transfus* OR infus* OR administ* OR requir* OR need* OR product* OR component* OR concentrate* OR apheres* OR pooled OR "single donor" OR "random donor")) |
| #3 | TS=(thrombocytopheres* OR plateletpheres*) |
| #4 | TS=((platelet* OR thrombocyte*) NEAR/5 (protocol* OR trigger* OR threshold* OR schedul* OR dose* OR dosing OR usage OR utilisation OR utilization)) |
| #5 | #1 OR #2 OR #3 OR #4 |
| #6 | TS=((("low birthweight" OR "low birth weight" OR VLBW OR ELBW OR Prematur* OR Preterm OR Pre-term) NEAR/2 (Infant* OR Neonat* OR newborn* OR new-born* OR baby* OR babies)) OR "neonatal intensive care" OR NICU) |
| #7 | #6 AND #5 |
| **Scopus** | |
| **Number** | **Query** |
| #1 | TI=((platelet* OR thrombocyte*) AND transfus*) |
| #2 | TITLE-ABS-KEY((platelet* OR thrombocyte*) pre/5 (prophyla* OR transfus* OR infus* OR administ* OR requir* OR need* OR product* OR component* OR concentrate* OR apheres* OR pooled OR "single donor" OR "random donor")) |
| #3 | TITLE-ABS-KEY(thrombocytopheres*  OR  plateletpheres*) |
| #4 | TITLE-ABS-KEY((platelet* OR thrombocyte*) pre/5 (protocol* OR trigger* OR threshold* OR schedul* OR dose* OR dosing OR usage OR utilisation OR utilization)) |
| #5 | #1 OR #2 OR #3 OR #4 |
| #6 | TITLE-ABS-KEY((("low birthweight" OR "low birth weight" OR VLBW OR ELBW OR Prematur* OR Preterm OR Pre-term) pre/2 (Infant* OR Neonat* OR newborn* OR new-born* OR baby* OR babies)) OR "neonatal intensive care" OR NICU) |
| #7 | #6 AND #5 |

**Suppl2: PRISMA flow diagram of the studies’ screening and selection**

**Suppl3: Demographic and Intervention data in studies comparing preterm infants who received platelet transfusion vs. non-transfused infants**

| Author, year | Study design | N | n | Male  n (%) | Gestational age, weeks  (mean ± SD) | Birthweight,  grams  (mean ± SD) | Intervention details |
| --- | --- | --- | --- | --- | --- | --- | --- |
| Andrew,  1993 [14-16] | RCT | 152 | **G1:** 78 | 49 (62.8) | 27.4 ± 2.2 | 915 ± 235 | Range: 1-3 PTx per infant  Goal: maintain the platelet count >150x10^9^/L until day 7 of the study |
|  |  |  | **G2:** 74 | 40 (54.1) | 27.7 ± 2.5 | 931 ± 266 | No PTx were given, unless the platelet count fell to <50x10^9^/L or the infant was bleeding |
| Christensen, 2006 [17] | Retrospective cohort | 283 | **G1:** 129 | - | - | - | Total number of transfusions: 357  Range: 1-51 PTx per infant |
|  |  |  | **G2:** 154 | - | - | - | No PTx were given |
| Baer, 2007 [18] | Retrospective cohort | 1600 | **G1:** 494 | 283 (57.3) | 30.9 ± 5.7 | 1518 ± 1106 | Range: 1-62 PTx per infant  Transfusion criteria:  - PC<100 x10^9^/L in ECMO or immediate pre/post-surgery  - PC<50 x10^9^/L in unstable patients - PC<20 x10^9^/L in stable patients |
|  |  |  | **G2:** 1106 | 653 (59.0) | 35.0 ± 4.4 | 2384 ± 991 | No PTx were given |
| Bonifacio,  2007 [19] | Nested  Case-control | 82 | **G1:** 60 | - | 25.7 ± 2.2 | 762 ± 291 | PTx were given to the 60 patients with moderate and severe thrombocytopenia in the study |
|  |  |  | **G2:** 22 | - | 28.7 ± 2.6 | 1158 ± 477 | No PTx were given |
| Sparger,  2016 [20] | Retrospective cohort | 972 | **G1:** 231 ^a^ | 141 (61.0) | 26.3 ± 3.0 | 805 ± 284 | Total number of transfusions: 1002  Mean number of transfusions ± SD: 4.3 ± 6.0  Range: 1-63 PTx per infant |
|  |  |  | **G2:** 741 ^a^ | 379 (51.1) | 28.8 ± 2.6 | 1113 ± 264 | No PTx were given |
| Alhamad,  2022 [21] | Retrospective cohort | 154 | **G1:** 77 | 38 (49.4) | 34-36: 8 (10.4) ^b^ 32-33: 4 (5.2) ^b^ 28-31: 31 (40.3) ^b^  <28: 34 (44.2) ^b^ | <2500: 5 (6.5) ^c^ <1500: 22 (28.6) ^c^ <1000: 49 (63.6) ^c^  >2500: 1 (1.3) ^c^ | Total number of transfusions: 296  Most infants received from 1 to 3 PTx |
|  |  |  | **G2:** 77 | 38 (49.4) | 34-36: 8 (10.4) ^b^ 32-33: 4 (5.2) ^b^ 28-31: 31 (40.3) ^b^  <28: 34 (44.1) ^b^ | <2500: 13 (16.9) ^c^ <1500: 36 (46.8) ^c^ <1000: 28 (36.4) ^c^  >2500: 0 (0.0) ^c^ | No PTx were given |
| Chen, 2022 [22] | Retrospective cohort | 1221 | **G1:** 94 | 56 (59.6) | 28.0 (27.0-30.0) ^d^ | 1060 (930-1290) ^d^ | Total number of transfusions: 166  Range: 1-8 PTx per infant |
|  |  |  | **G2:** 1127 | 675 (59.9) | 31.0 (29.0-33.0) ^d^ | 1600 (1305-1980) ^d^ | No PTx were given |
| Raja, 2022 [23] ^e^ | Retrospective cohort | 540 | **G1:** 105 | - | 26.4 ^f^ | 760 ^f^ | Mean threshold for PTx ± SD:  62.190 x 10^9^/L ± 27.100 x 10^9^/L |
|  |  |  | **G2:** 435 | - | - | - | No PTx were given |

Abbreviations: N, total number of infants in the study; n, number of infants per group; SD, standard deviation; G1, Group 1 – received platelet transfusion; G2, Group 2 – did not receive platelet transfusion; PTx, platelet transfusion; PC, platelet count; ECMO, extracorporeal membrane oxygenation; RCT, randomized controlled trial. (-): Missing data

^a^ Number in patient-days, during the first 7 days of life with a platelet count <100 x 10^9^/L, in whom the outcomes were studied: 212 in group 1 and 190 in group 2

^b^ Gestational age range in weeks: number of infants (%)

^c^ Birthweight range in grams: number of infants (%)

^d^ Median (interquartile range)

^e^ Abstract only-publication – some data not available

^f^ Unknown standard deviation

**Suppl4: Risk of bias assessment in the included randomized controlled trials, using the Cochrane risk-of-bias tool, version 2** [11]


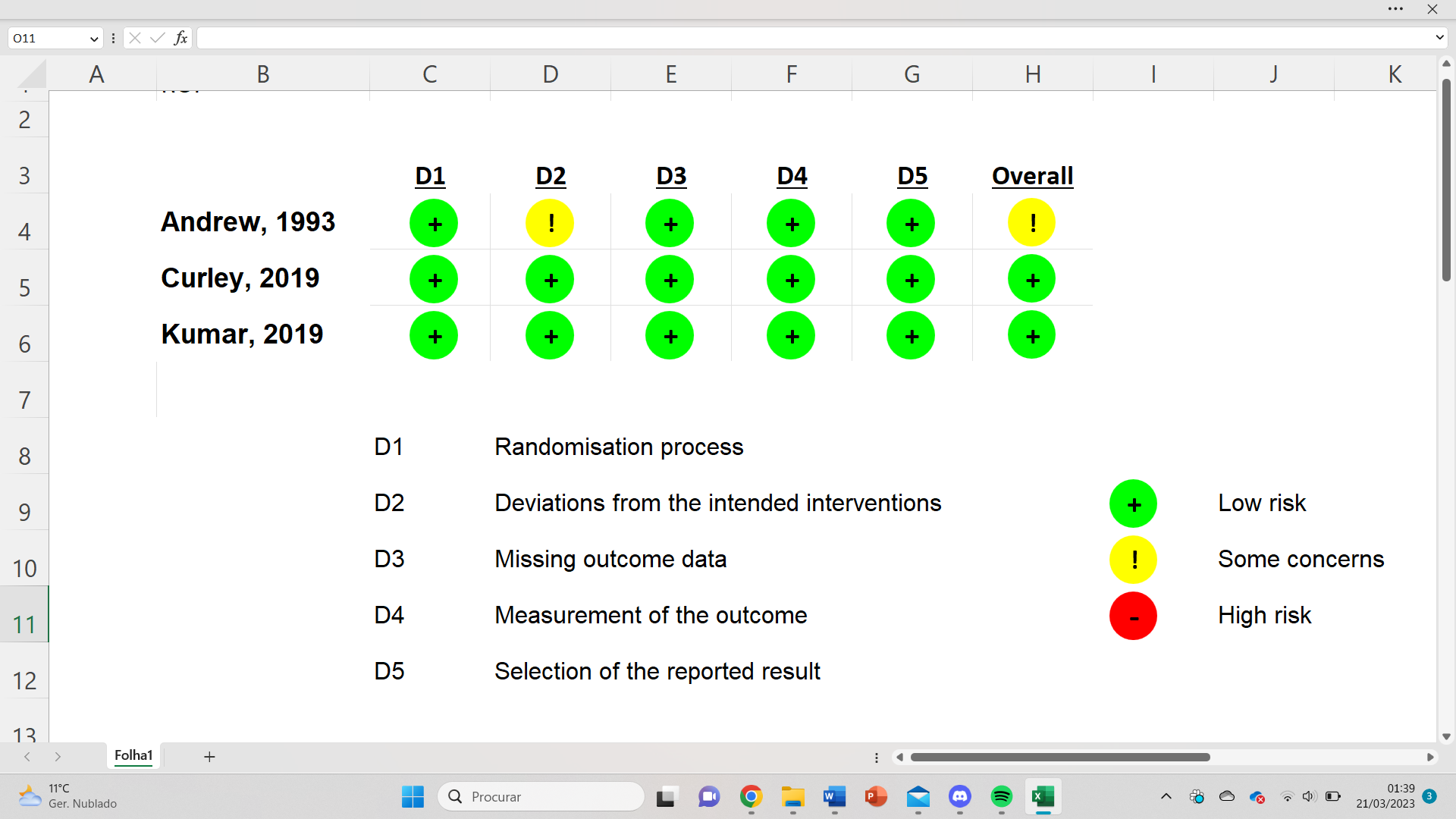


**Suppl5: Risk of bias assessment in the included cohort and case-control studies, using the Newcastle-Ottawa Scale** [13]


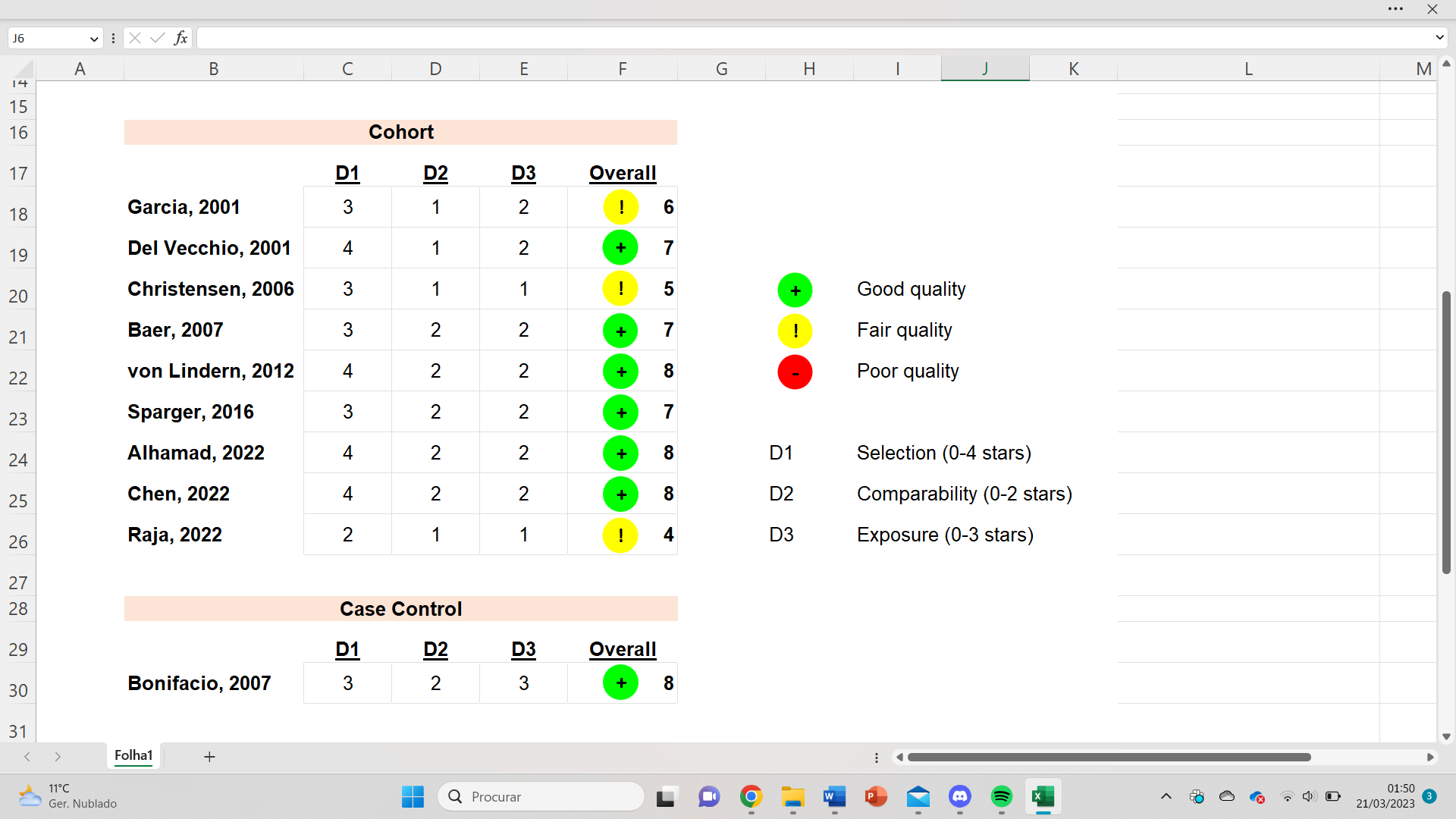


**Suppl6: Leave-one-out sensitivity analysis for mortality**

|  | RR | 95% CI | p-value | tau^2^ | tau | I^2^ |
| --- | --- | --- | --- | --- | --- | --- |
| Omitting Christensen, 2006 [17] | 3.6 | [1.9; 6.9] | < 0.0001 | 0.3304 | 0.5748 | 82.4% |
| Omitting Baer, 2007 [18] | 2.4 | [1.8; 3.4] | < 0.0001 | 0.0286 | 0.1692 | 12.0% |
| Omitting Bonifacio, 2007 [19] | 3.3 | [1.8; 5.6] | < 0.0001 | 0.3160 | 0.5622 | 82.3% |
| Omitting Alhamad, 2022 [21] | 3.6 | [1.9; 6.9] | 0.0001 | 0.3460 | 0.5882 | 83.0% |
| Omitting Chen, 2022 [22] | 3.1 | [1.5; 6.3] | 0.0022 | 0.4405 | 0.6637 | 86.8% |

Abbreviations: RR, relative risk; CI, confidence interval

**Suppl7: Leave-one-out sensitivity analysis for sepsis**

|  | RR | 95% CI | p-value | tau^2^ | tau | I^2^ |
| --- | --- | --- | --- | --- | --- | --- |
| Omitting Baer, 2007 [18] | 2.7 | [1.0; 7.3] | 0.0555 | 0.5991 | 0.7740 | 86.7% |
| Omitting Bonifacio, 2007 [19] | 4.5 | [3.7; 5.6] | < 0.0001 | 0.0000 | 0.0000 | 0.0% |
| Omitting Alhamad, 2022 [21] | 3.1 | [1.3; 7.7] | 0.0116 | 0.5667 | 0.7528 | 91.0% |
| Omitting Chen, 2022 [22] | 2.5 | [1.0; 6.2] | 0.0428 | 0.4807 | 0.6933 | 90.2% |

Abbreviations: RR, relative risk; CI, confidence interval
